# An assessment of the nutritional intake of soccer referees

**DOI:** 10.1186/s12970-015-0068-9

**Published:** 2015-02-07

**Authors:** Cristian Martínez Reñón, Pilar S Collado

**Affiliations:** Faculty of Sports Sciences (FCAFD), University of Leon, Leon, Spain; Institute of Biomedicine (IBIOMED), University of Leon, Leon, Spain; Department of Biomedical Sciences. Physiology, University of León, 24071 León, Spain

**Keywords:** Nutritional requirements, Dietary record, Soccer referees, Macro-nutrients, Analyses

## Abstract

**Objective:**

The present study aims to analyze the eating habits and attitudes of a group of soccer referees and linesmen.

**Method:**

A nutritional study was undertaken of thirty-five soccer referees (aged between 18 and 50) refereeing at different levels, from the Spanish national third division down to the provincial second division. Through the use of a 3-day food diary and 24-hour recall, this study analyzed the intake and distribution of macro- and micro-nutrients and of dietary fiber consumed on different types of day (normal, training, and match days).

**Results:**

There were no significant differences in calorie intake related to the three types of day (normal, training, and match days). This was true both of overall amounts (2371.1 kcal, 2479.7 kcal, and 2368.4 kcal, respectively) and amounts per unit of body weight (32.4 kcal/kg, 33.9 kcal/kg, and 32.4 kcal/kg, respectively). In respect of macro-nutrient intake, more specifically carbohydrates, the subjects consumed a diet with an insufficient amount of carbohydrates: 279 g, as against the 371 g (REC1) or 540 g (REC2) recommended according to physical activity levels. Slight increases were observed on game days, but were not statistically significant. Consideration of micro-nutrients showed that the quantities of three vitamins (B6, B12, and C) consumed were above the recommended amounts. However, this was not an issue, since the figures related to water-soluble vitamins. Finally, the amounts of minerals (Ca, Mg, and Fe) and fiber consumed were close to recommended values, regardless of the type of day being considered.

**Conclusions:**

This study found that the group of referees investigated consumed a diet that did not have sufficient calories from carbohydrates, in view of their occupation. This poor nutritional status might interfere with the development of their sporting performance and ultimately increase the risk of injury. This implies a need to design and implement a diet and to introduce educational programs on nutrition for these sportspeople.

## Introduction

The link between nutritional habits and health has concerned mankind since the origin of the earliest societies and cultures. Scientific study of the relationship between eating habits and health began in the eighteenth century, when long sea journeys led sailors to have poor and monotonous diets that caused deficiency diseases [1]. Today, this concern is made evident by the increasing demand for nutritional information related to sport and physical activity. Interest in this matter is found in many areas, from elite athletes who are eager to win medals in the Olympics or other championships, down to individuals who practice sport simply to keep fit [2].

There is no such thing as a complete, perfect, and magical food [3]. The ideal formula would be based on choosing a variety of foodstuffs that, when combined, would provide a balance for the proper functioning of the metabolism. This balance must be adequate to meet the demands of concerted effort, for instance, a soccer game.

It is well known that food intake influences an athlete’s training and thus, ultimately, his or her performance levels. For this reason, sportspeople who want to achieve a higher performance, regardless of their specialties, ought to be interested in their own nutrition. Sports performance is linked to the proportions of basic nutrients in the daily diet. The attention that athletes pay to their diet should go beyond the day of a competition or match; their dietary concerns must embrace every day of their lives. It is true, nevertheless, that the food eaten before a competition or game merits particular attention [4].

Nowadays, there are organizations, for example the World Health Organization (WHO), that are firmly committed to evaluating different diets that are appropriate to specific areas. Their work informs the general population about these findings. Furthermore, since energy requirements differ for each sport, there are also many publications that consider the most appropriate nutritional recommendations for optimizing athletes’ performance levels in such areas as getting the greatest benefit from training, improving recovery, or maintaining ideal weight and fitness, thus minimizing the risk of injury and illness [5]. However, there is still little specific literature related to nutrition for certain groups.

In spite of the fact that it affects all sporting groups and disciplines, sports nutrition has not received widespread attention in the literature. The group referred to in the present study was soccer referees and linesmen. Nevertheless, interest in nutrition with a view to improving performance in soccer in general is relatively recent [6,7]. Soccer is one of the most important sports worldwide. Although it is the players who attract the most attention in the soccer world, referees, linesmen, and other officials are of equal importance. As in all other sports, these individuals require an optimal fitness level so that they can react to the demands of the game in a satisfactory manner. Hence and as is the case with any other sport, it is necessary to consider the effects of certain aspects of their nutritional intake on their performance. Similarly to the demands made of soccer players, refereeing is characterized by cycles of short, high-intensity bursts of activity, essentially sprinting, combined with lower-intensity physical effort, such as medium- or light-intensity jogging [8]. In addition, attention must be paid to the category of soccer (in the sense of teams’ playing standards) that each referee and linesman officiates, since physical and nutritional requirements will vary as categories change from less demanding (second division at provincial level) to more demanding (third division at national level).

Another factor that must be considered is the great distances that are run in a discipline such as soccer. Numerous studies that make use of GPS technology have shown that during soccer match participants travel distances of around 11,000 meters [9]. In soccer and in team sports in general, one of the most important factors in athletic performance is recovery from fatigue after training and competitions or games. This is especially the case for sports in which participants sometimes train and compete on the same day or on successive days, with little recovery time. The recovery process is influenced by numerous elements. One of the most important of these is nutrition, but its effectiveness depends on many variables, including the competition itself and the sex, training status, and nutritional status of the subjects [8,10].

In view of all the evidence discussed above, the present study aims to analyze the eating habits and attitudes of a group of soccer referees, and in so doing seeks to address the shortage of studies in the literature that investigate these aspects of referees’ diets and compare them to recommended patterns. The intention in conducting the study was to demonstrate the importance of proper nutrition, both in general and for sportspeople at a particular level. More specifically, the aim was to compare the nutritional profiles of the referees studied with the values from two different sets of recommendations (aimed at sedentary individuals and high-level athletes), and also to assess the nutritional intake and eating patterns of referees according to the type of day (normal day, training day, or match day).

## Methodology

### Subjects

A qualitative descriptive epidemiological study of 35 soccer referees of different categories (from Spain’s third division at national level down to the second division at a provincial level, roughly equivalent to county level) was undertaken. The inclusion criteria for the study included: being a soccer referee within one of the previously described categories; being 18 or older; and being free of injury at the time of the study. All subjects who participated in the study were active referees who had undergone a medical examination by their sports federation. Those taking any medication at the moment of the study were excluded. The average age of subjects was 25.9 years. All the referees had a day job that they combined with their refereeing duties. Participants were informed of the objective of the study in a talk that was given in order to explain what was going to be done, and they all submitted a signed consent form that had been specially prepared for the study. They were given the assurance that they could drop out of the study at any time should they so wish. The study followed the principles of the Declaration of Helsinki, and the local Ethics Committee of the University of Leon approved all procedures.

### Dietary assessment

The subjects compiled a food diary, using forms provided for that purpose. These forms required them to record information about what they consumed each day: amounts, proportions of ingredients, methods of preparation, and so forth [11]. Each of these forms had an initial guidelines section to assist participants throughout the process, which lasted three days. Photographs of food models, measuring spoons, cups, bowls, and other prompts were called on to further improve the quality of the collected dietary data. Once the subject returned the 3-day food record, a trained researcher immediately checked through it and followed up with the athlete to request clarifications about specific items and/or obtain more detail if necessary.

All of the subjects in the group were also interviewed personally, by a trained research assistant, 24 hours they had submitted their forms. The aim of this interview was to rerecord the same information provided by the individual dietary records in order to perform a cross-check and thereby make the data used in the study more reliable.

The information about food, quantities, batches, and cooking processes collected from the participants by means of the dietary records and 24-hour recalls was manually analyzed on a daily basis and inputted. All the data were processed by the Nutriber V.1.1.1.R5 software package (produced by Funiber, Barcelona, Spain) in order to calculate individual diets. The combination of food diaries and recall after a twenty-four-hour period met the criteria for a simple, rapid, and inexpensive research method.

Determination of basal metabolism was by means of the Harris-Benedict equation [12]. This has been demonstrated to have good reliability and applicability in a number of studies [13]. For males, such as the participants in this study, the formula is:

$$ 66.473 + \left( 13.7516 \times weight\  in\; kilograms\right) + \left( 5.0033 \times height\  in\; centimeters\right) - \left( 6.755 \times age\  in\  years\right) $$

### Statistical analysis of the data

A Microsoft Office Excel 2007 spreadsheet was used to record and handle data and to produce graphs. The results were reported as mean values and standard error of the mean. In working out the Student’s T-test of the sample, the statistical software package SPSS 17.0 was used. Values were taken as significant with p <0.05 and highly significant with p <0.01.

## Results

Table 1 shows the characteristics of participants and their kilocalorie intakes and basal metabolic rates. Like the work done by Martínez Reñón et al. [14], in this nutritional study the sample was compared with the tables of recommended energy and nutrient intakes (RDI: Recommended Daily Intake) for the Spanish population as a whole [1]. Specifically, it was compared with two recommendations: REC1 (2,700 Kcal), for people of the same age and sex as the referees who undertake only light activity, and REC2 (3,600 Kcal), for people of the same age and sex as the referees who practice similar sports that require the same high levels of activity [1].

**Table 1:** **General characteristics**

**Age**	24.7 ± 9.9 years.
**Height**	178.5 ± 6.5 centimeters.
**Weight**	73.1 ± 8.2 kilograms.
**Average intake**	2408.8 ± 517.8 kilocalories.
**Normal day average intake**	2371.1 ± 519.1 kilocalories.
**Training day average intake**	2479.7 ± 430.7 kilocalories.
**Game day average intake**	2368.4 ± 584.7 kilocalories

An energy intake of 2,700 kcal is indicated by REC1 and one of 3,600 kcal by REC2. However, the total average energy intake of the subjects was 11% less than the first (the recommended intake for people with light activity), and 33.1% lower than the second (the recommendation for professional athletes of a similar age). The subjects were thus well below recommended energy intakes.

Calorie and macro-nutrient intakes are shown in Table 2, classified according to the type of day: normal (no training, with referees going about their usual trade or profession), training (which can possibly happen alongside continuing their normal occupations), and match days. The results show that calorie intakes did not vary from one type of day to another.

**Table 2:** **Mean macro-nutrient intake values on the different types of day ± standard error of mean**

	**Normal**	**Training**	**Match**
**Energy**			
**Kcal**	2371.1 ± 519.1	2479.7 ± 430.7	2368.4 ± 584.7
**Kcal/kg**	32.4 ± 7.6	33.9 ± 6.9	32.4 ± 5.3
**Carbohydrates**			
**g**	**278.9 ± 86.2**	**291.2 ± 54.0**	**300.6 ± 84.0**
**Kcal**	1115 ± 181	1164 ± 129	1202 ± 195
**%**	47.0	46.9	50.7
**Proteins**			
**g**	**125.8 ± 17.3**	**115.2 ± 40.0**	**118.5 ± 37.0**
**Kcal**	503.2 ± 178	460.8 ± 138.4	474.0 ± 113.6
**%**	21.2	18.5	20.0
**Fats**			
**g**	**100.2 ± 62.0**	**110.1 ± 35.0**	**97.6 ± 83.0**
**Kcal**	918.0 ± 121.6	990.1 ± 181.1	878.4 ± 161.9
**%**	38.7	39.8	36.8

**Figure 1:**
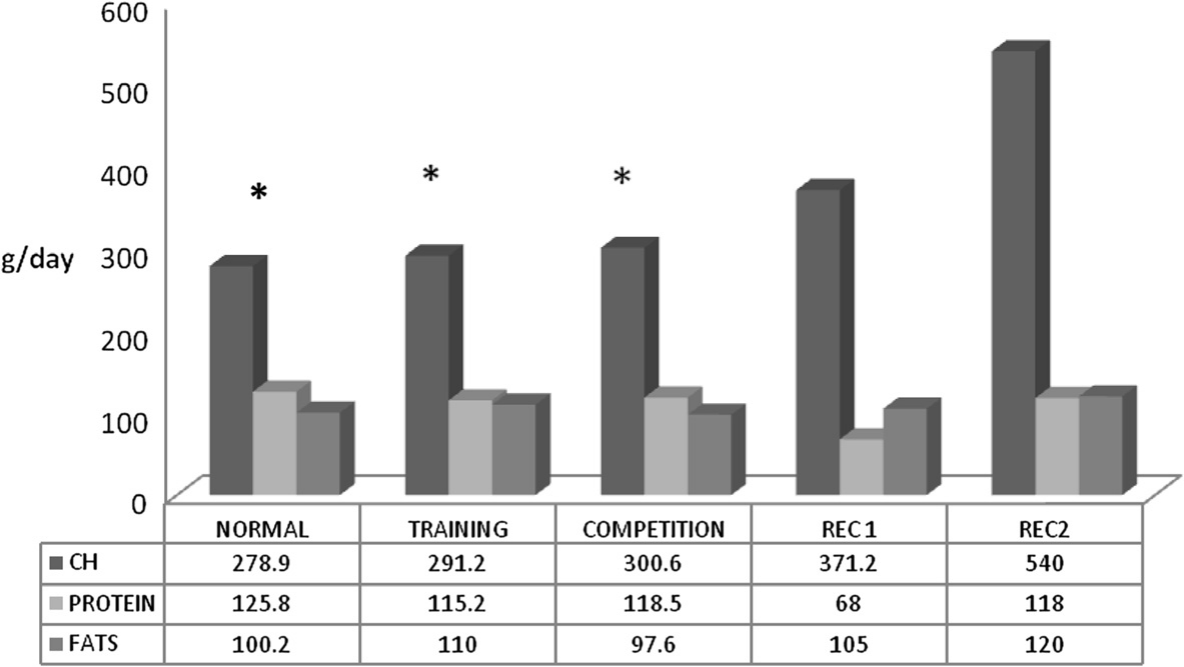
**Relationships between mean values for macro-nutrient intakes and recommendations, depending on the type of day.** *(p < 0.001 versus REC2). REC1 = Recommendation for a person of the same age and sex undertaking light activity. REC2 = Recommendation for a person of the same age and sex undertaking a high level of activity.

Figure 1 shows that relative to REC1 and REC2 the subjects’ diets were low in carbohydrates, averaging 279 g, against the 371 g of REC1 and the 540 g of REC2. Similarly, they were high in protein, with an average of 125.8 g, compared to the 62.8 g of REC1 and 117.9 g of REC2. However, as can be seen in Table 2 and Figure 1, the type of day did have a small effect on carbohydrate intake, with a slight increase on match days. Nevertheless, this intake increase did not reach the values of either of recommendations REC1 and REC2.

Finally, with regard to fats, there were no significant differences from either of the recommendations, although the values noted did exceed the 30% of the total calorie intake suggested by REC1. There were no differences that were dependent on the type of day in the percentage values, which were 38.7%, 39.8% and 39.3% for normal, training, and match days respectively.

With regard to micro-nutrients, the vitamins that were assessed were B1, B2, B6, B12 and C. As with the macro-nutrients, intakes of vitamins and minerals were compared to RDI values. However, there was no need to make two separate comparisons with REC1 (low activity) and REC2 (high-performance athletes), since the recommended values for micro-nutrients are the same for both these populations (Table 3). Vitamin intake was therefore compared to REC1, and then subdivided according to the type of day involved.

**Table 3:** **Relationship between mean values of intake of vitamins, minerals and fiber and its recommendation**

		**Total**	**Days**			**Recommendation**
			**Normal**	**Training**	**Competition**	
Vitamins (mg)	B1	2.1±0.6	2.1±0.7	1.9±0.8	2.4±2.0	1.2
	B2	2.2±0.4	2.2±0.7	2.2±0.8	2.3±1.0	1.8
	B6	2.4±0.8*	2.6±1	2.3±0.9	2.2±0.9	1.1-1.8
	B12	16.9±1*	16.0±1.5	15.6±1.9	19.3±2.1	2
	C	141±11*	170±15	135±28	116±21	60
Minerals (mg)	Ca	1138±41	1161±44	1198±89	1057±149	800-1000
	Mg	376±122	389±151	383±126	356±80	350-400
	Fe	52.3±8.2*	50.1±12.7	62.4±16.1	44.5±5.4	10-15
Fiber (g)		23.3±2.4	22,2±2,7	28,5±3,8	19,2±3,1	25

*( p<0, 05 versus recommendation).

The intake of B-complex vitamins (B1, B2, B6, and B12) can be seen from Table 3 to have been relatively high compared to the recommendations.

Of the individual vitamins, B6 showed a marked increase, at more than 50% higher than the recommended amount (p < 0.05). Most striking was vitamin B12, with stratospheric values of up to eight times the daily amount recommended for the “slightly active” population being recorded. Vitamin C intake was 60% higher than recommended total values.

Consideration of vitamin intakes according to the type of day showed that there were no significant differences among them. One interesting point was the figure for vitamin B12 on match days. This amounted to 19.3 milligrams, a level much higher than the recommended value.

The minerals analyzed were Ca, Mg, and Fe. The data for them are shown as milligrams (Table 3).

On average, the totals for all these minerals were either higher than recommended values or within the recommended range. For example, the mean amount of calcium (Ca), an essential mineral for bone production, lay close to the recommended range of 800 mg to 1,000 mg. The test subjects had an average daily intake of 1,138 mg of Ca, a figure somewhat on the low side, since the sample population was one performing moderate- to high- intensity activities.

The values for the intake of magnesium (Mg) were very similar to the quantities laid down in REC1 and REC2. In contrast, figures for iron (Fe) were more than 60% higher than the amounts suggested by REC1 and REC2.

A check on the quantities of minerals depending on the type of day showed no data of importance. This was because no differences were found between one type of day and another.

The data on fiber intakes recorded were very similar in terms of total amounts. The average quantity noted was 23.3 g, where recommended values would be around 25 g of fiber daily. When the figures were broken down by type of day, the differences were not statistically significant, all of them being close to the recommended amounts. Nonetheless, it is noteworthy that fiber intakes on training days averaged 28.5 g, while on normal days they were 22.3 g on average, and on match days the average fell to 19.2 grams.

## Discussion

The importance of nutrition in professional refereeing, as in any sports activity, would seem to be more than clear. The nutritional requirements for each sport are specific and are related to the energy demands that the sport in question imposes [15]. The type, intensity, and duration of exercise affect the use of energy substrates. Thus, to satisfy the nutritional needs for different sports, in this case soccer it is necessary to develop adequate dietary plans. As was established by previous research, the requirements of a soccer referee are similar to those of a soccer player [16], and an adequate diet will positively influence the performance of these sportspeople. There is therefore a need to adjust the consumption of foods in the daily life of soccer referees so that they are be able to choose the right food at the right time in order to be efficient [17].

The importance of this can be seen in the fact that sportspeople who fail to observe these requirements may suffer an increased risk of injuries [18] and decreased performance [11]. It has been reported that approximately 25% of injuries occur in the last 15 to 20 minutes of a game, and this is because at that point energy reserves are coming to an end [19]. Nutritional management is therefore essential to achieve maximum performance in the practice of refereeing. There is widespread evidence that adjusting energy intake to energy expenditure is essential. The calorie intake of participants (kcal) in the present study was determined to be 2408 ± 518 kcal on average. This value is well below the recommendations established for sportspeople of the same age, sex, and level of activity, with the difference even more evident when compared with the requirements at a professional level. As already mentioned, an insufficient energy intake such that as found in the participants in the study can lead to a decrease in performance or an increase in the risk of injuries.

The results obtained were separated according to the type of day (normal, training, and match days). The participants kept their calorie-consumption levels more or less steady over the three sorts of day. Nonetheless, when the amount of macro-nutrients was considered, it was noticed that there was a slight increase, though not a significant upturn, in the consumption of carbohydrates on match days. It is thus evident that the intake of this type of nutrient did not conform to the recommendations. This should be regarded as an aspect requiring correction.

The recommended daily consumption of carbohydrates to maintain muscle glycogen stores over several days of intense training is between 500 g and 600 g, that is, between 60% and 70% of total energy intake [20]. However, in this study the amount of carbohydrates eaten fell well below these recommendations, to 33% less than the recommendation for athletes (REC2) and even to 11% less than the recommended intake for the group undertaking only light activity (REC1). A similar result was recorded in previous studies involving semi-professional soccer players, who were found to consume 40% less than the REC2 recommendation [14]. There is widespread evidence to suggest that an increase in carbohydrate intake can improve performance in various kinds of sports activity, including refereeing, which requires intermittent running at various intensities. Sportspeople who consume a diet rich in carbohydrates were found to be able to undertake 33% more high-intensity running during a game, and a diet with 65% carbohydrates was observed to improve performance when compared with alternatives in which carbohydrate consumption amounted to only 30% of the total diet [21-23]. A super-consumption of carbohydrates has shown noticeable benefits. However, this fact was not known to any of the participants [21-23].

When carbohydrates were analyzed by type of day, no significant differences were observed, with very similar carbohydrate intake values being obtained on all three sorts of day. One possible explanation for these results may be that the subjects did not have a good basic understanding of nutrition, and as a result their diet did not meet their daily needs.

Standard recommendations for the consumption of proteins are 0.80 g/kg/day. However, it is fully accepted that these amounts should be increased to between 1.4 g/kg/day and 1.7 g/kg/day, or in other terms 98 g/day to 119 g/day of proteins, for people who participate in intense physical activities [24]. The referees and linesmen in the sample had a greater consumption of proteins than the recommendation for adults, at 125.8 g/day. This may be of benefit to their performance, as the demands in relation to stamina and strength made upon them by their activity may be favorably affected by a protein intake that is above recommendations. This level of consumption may improve strength and provide amino acids to serve as a substrate for any increase in amino acid oxidation that may occur during training and games [25,26].

Finally, the preferred distribution of macro-nutrients indicates that the oils and fats consumed should provide between 20% and 30% of total calorie intake, even for sportspeople. In the present study all the referees’ food diaries showed consumption falling within the recommended values. Although this type macro-nutrient helps to meet the increased energy demands of strenuous exercise, an increased consumption of lipids would not appear to be necessary [24].

With regard to micro-nutrient analysis, it is important to note that vitamins are structurally related organic compounds, but differ in their physiological action. However, they were studied together because their presence in the diet is essential for the body to carry out specific metabolic reactions [2].

In respect of vitamins and exercise, there appear to be no studies supporting vitamin supplementation for any kind for sports. This would make sense only in cases of vitamin deficiency caused by an unbalanced diet or some particular metabolic disorder. The vitamins investigated were the B-vitamin complex and vitamin C. Consumption similar to recommended daily amounts was noted for vitamins B1 and B2. In the case of vitamins B6, B12, and C, values above the recommended amounts were recorded. There were no significant differences when vitamins were examined by type of day and activity. This provides a possible explanation of these high values in the participants: they may be due to high protein intake in the case of the vitamins found in animal proteins, or to the consumption of large amounts of energy drinks on certain days, as these contain considerable amounts of vitamin B6 and B12.

The results recorded for vitamin C showed a consumption that was double recommended daily amounts. Vitamin C, or ascorbic acid, is important for the formation and maintenance of collagen and the synthesis of neurotransmitters [27]. The reason for these enhanced quantities of vitamin C may be that participants consumed foods with larger amounts of it in the hope of avoiding catching colds. Water-soluble vitamins like vitamin C are stored in the body to only an extremely limited extent, and are easily removed from it because of their solubility, meaning that excessive consumption is rarely dangerous.

In respect of other micro-nutrients in the study, the minerals investigated were sodium (Na), calcium (Ca), magnesium (Mg), and iron (Fe); consumption of all of these was indicated in milligrams. The average amounts for the minerals calcium and magnesium were close to those recommended for the population in general or even for those facing few physical demands, with calcium in the sample showing approximately recommended levels. In the case of iron, the values recorded were much higher than recommended. One of the possible explanations for the high levels of iron may be that this mineral is found in many of the foods that were recorded as being consumed in appreciable quantities, for example meat.

In conclusion, it may be stated that the information collected in this study provides a brief look at the eating habits and nutritional status of referees and linesmen, a little-studied group whose members are nevertheless are of great importance in sports, and who are increasingly required to have a high level of performance and fitness. The methodology employed is capable of extrapolation across various population sectors as a result of its wide applicability. It can be stated that the sportspeople studied had a nutritional status that was not entirely suited to their circumstances, as their diet contained fewer carbohydrates and more proteins than recommended. However, no serious nutrient deficiency was observed, indicating that their diet was varied. Education and awareness-raising about proper nutrient intakes is a day-to-day necessity for sportspeople of a certain level, whether in their own specific disciplines or in respect of others they may try where this need may become manifest [28]. Rigorous evidence relating to this aspect must be given special attention [29,30]. Because it is obvious that performance in training influences performance in competition, the implication is that there is a need to design and implement recommended diets and to introduce programs of education on nutrition for such sportspeople.
